# Supplementary figures and images for: Induction of Hibernation and Changes in Physiological and Metabolic Indices in Pelodiscus sinensis
Source: Biology (Basel). 2023 May 15;12(5):720. doi: 10.3390/biology12050720 (PMC10215499; doi:10.3390/biology12050720)

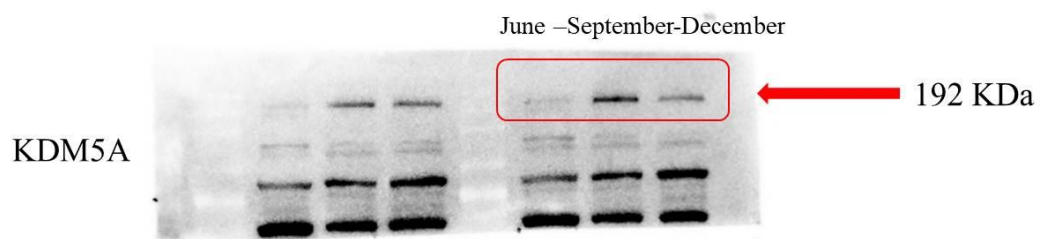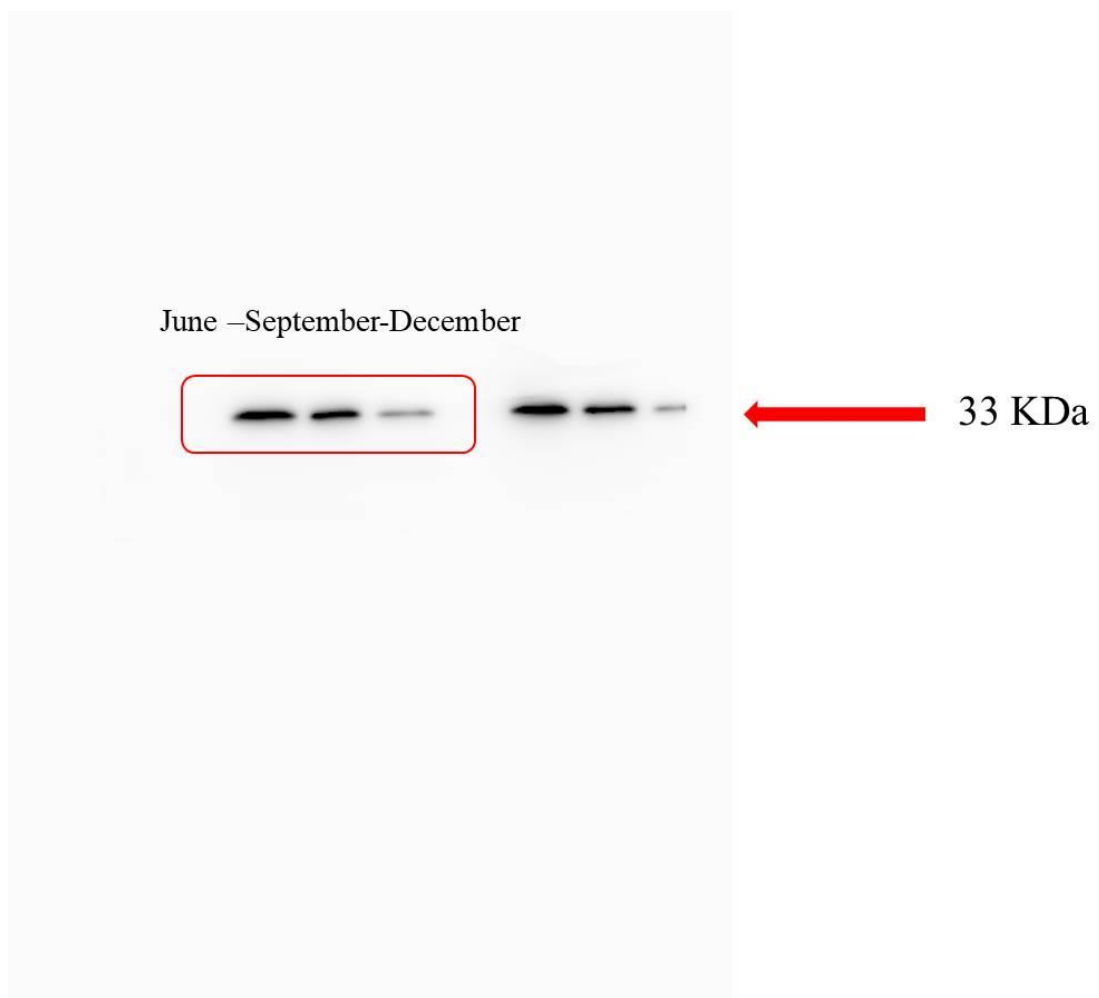

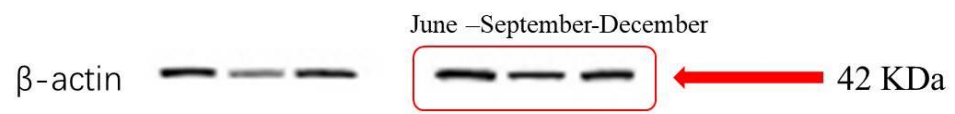

**Figure S1.** Full western blot of ASH2L and KDM5A.

Supplement: Supplementary file 1 [file biology-12-00720-s001.zip › biology-2366679-supplementary.pdf]
